# A randomised controlled, feasibility study to establish the acceptability of early outpatient review and early cardiac rehabilitation compared to standard practice after cardiac surgery and viability of a future large-scale trial (FARSTER)

**DOI:** 10.1186/s40814-023-01304-3

**Published:** 2023-05-11

**Authors:** Dumbor L. Ngaage, Natasha Mitchell, Alexandra Dean, Alex Mitchell, Sebastian Hinde, Enoch Akowuah, Patrick Doherty, Simon Nichols, Caroline Fairhurst, Kate Flemming, Catherine Hewitt, Lee Ingle, Judith Watson

**Affiliations:** 1 Castle Hill Hospital, Hull University Teaching Hospitals NHS Trust, Castle Road Cottingham, Hull, UK; 2 Department of Health Sciences, University of York, ARRC Building, York, UK; 3 Centre for Health Economics, Alcuin A Block, University of York, York, UK; 4 James Cook Hospital, South Tees Hospitals NHS Foundation Trust, Marton Road, Middlesbrough, UK; 5 Sport and Physical Activity Research Centre, Sheffield Hallam University, Collegiate Campus, Sheffield, UK; 6 Department of Sport, Health and Exercise Science, University of Hull, Hull, UK

**Keywords:** Cardiac surgery, Cardiac rehabilitation, Outpatient review, Feasibility, Median sternotomy

## Abstract

**Objective:**

To determine the acceptability and feasibility of delivering early outpatient review following cardiac surgery and early cardiac rehabilitation (CR), compared to standard practice to establish if a future large-scale trial is achievable.

**Methods:**

A randomised controlled, feasibility trial with embedded health economic evaluation and qualitative interviews, recruited patients aged 18–80 years from two UK cardiac centres who had undergone elective or urgent cardiac surgery via a median sternotomy. Eligible, consenting participants were randomised 1:1 by a remote, centralised randomisation service to postoperative outpatient review 6 weeks after hospital discharge, followed by CR commencement from 8 weeks (control), or postoperative outpatient review 3 weeks after hospital discharge, followed by commencement of CR from 4 weeks (intervention). The primary outcome measures related to trial feasibility including recruitment, retention, CR adherence, and acceptability to participants/staff. Secondary outcome measures included health-rated quality of life using EQ-5D-5L, NHS resource-use, Incremental Shuttle Walk Test (ISWT) distance, 30- and 90-day mortality, surgical site complications and hospital readmission rates.

**Results:**

Fifty participants were randomised (25 per group) and 92% declared fit for CR. Participant retention at final follow-up was 74%; completion rates for outcome data time points ranged from 28 to 92% for ISWT and 68 to 94% for follow-up questionnaires. At each time point, the mean ISWT distance walked was greater in the intervention group compared to the control. Mean utility scores increased from baseline to final follow-up by 0.202 for the intervention (0.188 control). Total costs were £1519 for the intervention (£2043 control). Fifteen participants and a research nurse were interviewed. Many control participants felt their outpatient review and CR could have happened sooner; intervention participants felt the timing was right. The research nurse found obtaining consent for willing patients challenging due to discharge timings.

**Conclusion:**

Recruitment and retention rates showed that it would be feasible to undertake a full-scale trial subject to some modifications to maximise recruitment. Lower than expected recruitment and issues with one of the clinical tests were limitations of the study. Most study procedures proved feasible and acceptable to participants, and professionals delivering early CR.

**Trial registration:**

ISRCTN80441309 (prospectively registered on 24/01/2019).

**Supplementary Information:**

The online version contains supplementary material available at 10.1186/s40814-023-01304-3.

## Key messages regarding feasibility

- What uncertainties existed regarding the feasibility? There was uncertainty regarding the feasibility of recruiting patients and surgical staff’s willingness to refer to early cardiac rehabilitation.
- What are the key feasibility findings? Participants were recruited over 8 months with retention at final follow-up being 74%, completion rates for outcome data time points ranged from 28 to 92% for ISWT and 68 to 94% for follow-up questionnaires, and at each time point, the mean ISWT distance walked was greater in the intervention group compared to the control.
- What are the implications of the feasibility findings for the design of the main study? These feasibility findings indicate that for any main trial, venue access would need improving, the recruitment process needs refining to increase the time interval for identifying and consenting patients, and early postoperative CPET would no longer be included.

## Introduction

Patients who have undergone cardiac surgery currently typically attend their first outpatient review 6 weeks after hospital discharge where their suitability to commence cardiac rehabilitation (CR) is determined before starting the programme from 8 weeks post-discharge. This time before commencement of CR does, however, extend a patient’s period of inactivity and requirement for management of surgery-related complications [1, 2]. Our prospective observational study (FORCAST6) found that although patients were satisfied with the 6 weeks interval, 44% would have liked an earlier review [3].

Following a median sternotomy, patients are told to refrain from lifting of heavy objects and other strenuous activities for 12 weeks to aid healing [4, 5]. This means that CR, along with the significant short- and long-term benefits that it provides after cardiac surgery [6], is delayed, diminishing its benefits [7] and potential for patients to recover physical fitness and activity capabilities [8]. Current guidelines for activity and exercise after sternotomy have been described as impeding patients’ recovery by being overly restrictive, and need for change is required [5].

Since sternal bone healing occurs by around 5 weeks [4], the rationale for the type and duration of such sternal precautions are unclear. Some research has shown that early patient review after hospital discharge reduces adverse outcomes following cardiac surgery [9]. In addition, the British Association for Cardiovascular Prevention and Rehabilitation (BACPR) Standards and Core Components [10], and the National Certification Programme for CR (http://www.cardiacrehabilitation.org.uk/NCP-CR.htm) both recommend starting CR early. Delaying CR commencement can prolong recovery, thereby increasing dependence on family and/or carers. The frustration caused by this may contribute to anxiety and depression reported in patients recovering from cardiac surgery [11].

This mixed-methods, randomised-controlled trial (RCT) therefore aimed to examine the feasibility of bringing forward outpatient review and CR to facilitate recovery, physical fitness and improve quality of life.

The study complied with the Declaration of Helsinki and was approved by East Midlands—Derby Research Ethics Committee on 10 January 2019 (Reference:18/EM/0391). Written informed consent was obtained from all patients and recruitment was open May 2019–December 2019.

## Methods

### Study design

This was a multicentre, open, feasibility RCT with embedded health economic and qualitative components. The methods are detailed in the published protocol [12] and summarised below.

### Patients and setting

Patients aged 18 to 80 years, who had elective or urgent cardiac surgery via a full median sternotomy from two participating hospital trusts in England were screened for eligibility.

### Intervention

All participants had a postoperative outpatient review, as per standard practice, to be certified fit to commence CR. This review occurred at 6 weeks post-hospital discharge in the control arm, and 3 weeks in the intervention arm.

The CR programme commenced at 8 weeks post-discharge in the control and 4 weeks in the intervention group. In both groups, CR consisted of supervised low-to-moderate intensity exercise performed once or twice a week for 8 weeks as per the centres’ standard practice. Exercise was prescribed according to standards published by BACPR [10].

### Study objectives

To determine the feasibility of delivering outpatient review 3 weeks post-discharge after cardiac surgery, followed by CR from 4 weeks. This included to:

- Assess surgical staffs’ willingness to conduct outpatient review 3 weeks after discharge and refer to CR.
- Examine patient enrolment barriers.
- Identify recruitment and attrition rates.
- Identify the most appropriate outcome measures.
- Test follow-up procedures and data collection tools.
- Assess the feasibility of conducting an economic evaluation for future definitive RCT.
- Gather outcome data to inform the sample size calculation for future definitive RCT.
- Inform any necessary redesign of a new recovery pathway in light of information gained.

### Sample size and randomisation

This was based on estimating a standard deviation for a potential primary outcome. The recruitment target of 100 participants allowed for a 30% attrition rate to still have 70 patients in the final analysis, as sample sizes between 24 and 70 have been recommended for feasibility trials in order to allow for the reliable estimation of a standard deviation [13]. Following surgery, consent, and completion of baseline data collection and assessments, authorised site staff randomised participants to either control or intervention groups using a remote, centralised service provided by York Trials Unit. The sequence was generated by a statistician not involved in the study. Participants were individually randomised and stratified according to the study site on a 1:1 basis using randomly-permuted blocks of varying sizes. Neither patients, healthcare staff nor members of the research team were blinded to trial allocation.

### Outcome measures

Demographics and EuroQoL-5 Dimensions 5 level (EQ-5D-5L) [14] were collected at baseline. At three post-randomisation time points (pre-CR, post-CR and 26 weeks post-randomisation), participants completed EQ-5D-5L and an NHS resource-use questionnaire.

Clinical data collected by local research nurses included: height, weight, body mass index, preoperative and post-operative details (baseline); heart rate, blood pressure, oxygen saturation at outpatient review and pre-CR; incremental shuttle walk test (ISWT) pre-CR, post-CR and at final follow-up; and cardiopulmonary exercise testing (CPET) at baseline and final follow-up at one study site only. Thirty and 90-day mortality, surgical site complications and hospital readmission rates were also collected.

In line with the objectives of a feasibility study, we also gathered information on:

i) Recruitment rates and drop-out to follow-up;
ii) Compliance to treatment arm allocation; and
iii) Acceptability of patient recruitment, early outpatient review and CR to patients, clinicians and NHS organisations.

### Impact of the COVID-19 pandemic

Due to the COVID-19 pandemic, from 18th March 2020, all outstanding participant final follow-up questionnaires (*n* = 28: 15 intervention; 13 control), were sent by post because of national lockdown rules, and clinical final follow-ups were completed by telephone. Furthermore, it was not possible to arrange a planned staff focus group to discuss difficulties encountered with recruitment, outpatient review, CR sessions and follow-up.

Interviews with participants highlighted how their activities had reduced due to limits on outdoor time, exercise venues closing and CR classes ceasing/not commencing.

### Qualitative interviews

Participants were invited to take part in two interviews; pre- and post-CR. From those agreeing, participants from both groups were purposively selected. Semi-structured telephone interviews were conducted to determine views on the timing of their outpatient review and readiness to commence CR.

All interviews were digitally recorded with permission, transcribed verbatim and analysed NVivo (version 12) coded and using thematic content analysis [15].

### Statistical analysis

A single analysis was undertaken at study end utilising Stata Version 16 [16]. Baseline data are summarised by group. Continuous variables are summarised using mean, standard deviation (SD), median, interquartile ranges (IQR) and range, while categorical data is reported as counts and percentages. Participant outcomes are summarised descriptively by group and time point, including the extent of missing data. No formal hypothesis tests were conducted due to the focus being on feasibility.

Recruitment rate was estimated along with a 95% confidence interval and summarised overall, by month and by site. Attendance at review appointments and CR sessions, and time between surgery and these events, are summarised by group. Questionnaire return rates are presented overall and by group and adverse events are summarised descriptively. The ISWT was summarised descriptively at each timepoint, with the standard deviation being estimated alongside an 80% confidence interval in line with current guidance on estimation of standard deviations from feasibility trials [17, 18]. Other outcomes collected at the end-of-study clinical follow-up are summarised descriptively by group. A post hoc summary of participants who had posted their final follow-up questionnaire early due to the COVID-19 pandemic is reported overall and by group.

### Economic analysis and quality of life data

Since this was a feasibility trial, a formal estimation of the cost-effectiveness of the respective interventions was not undertaken. Instead, the feasibility of collecting data needed for an economic analysis of a full-scale trial and exploration of the rate of response and missingness was considered. Estimates of patient benefit, determined from the EQ-5D-5L, and NHS resource-use, using patient-reported questionnaires, are summarised for the two groups.

### Patient and public involvement

A Patient Advisory Group held meetings with research nurses, CR staff and the chief investigator to discuss challenges encountered and potential solutions. The group acted as an important source of reference and the research progress was discussed. In the initial stages of the study, they assisted with study documentation development; identified barriers to participation and suggested possible solutions. They also contributed to the topic guides for the qualitative research.

## Results

### Recruitment

**Fig. 1:**
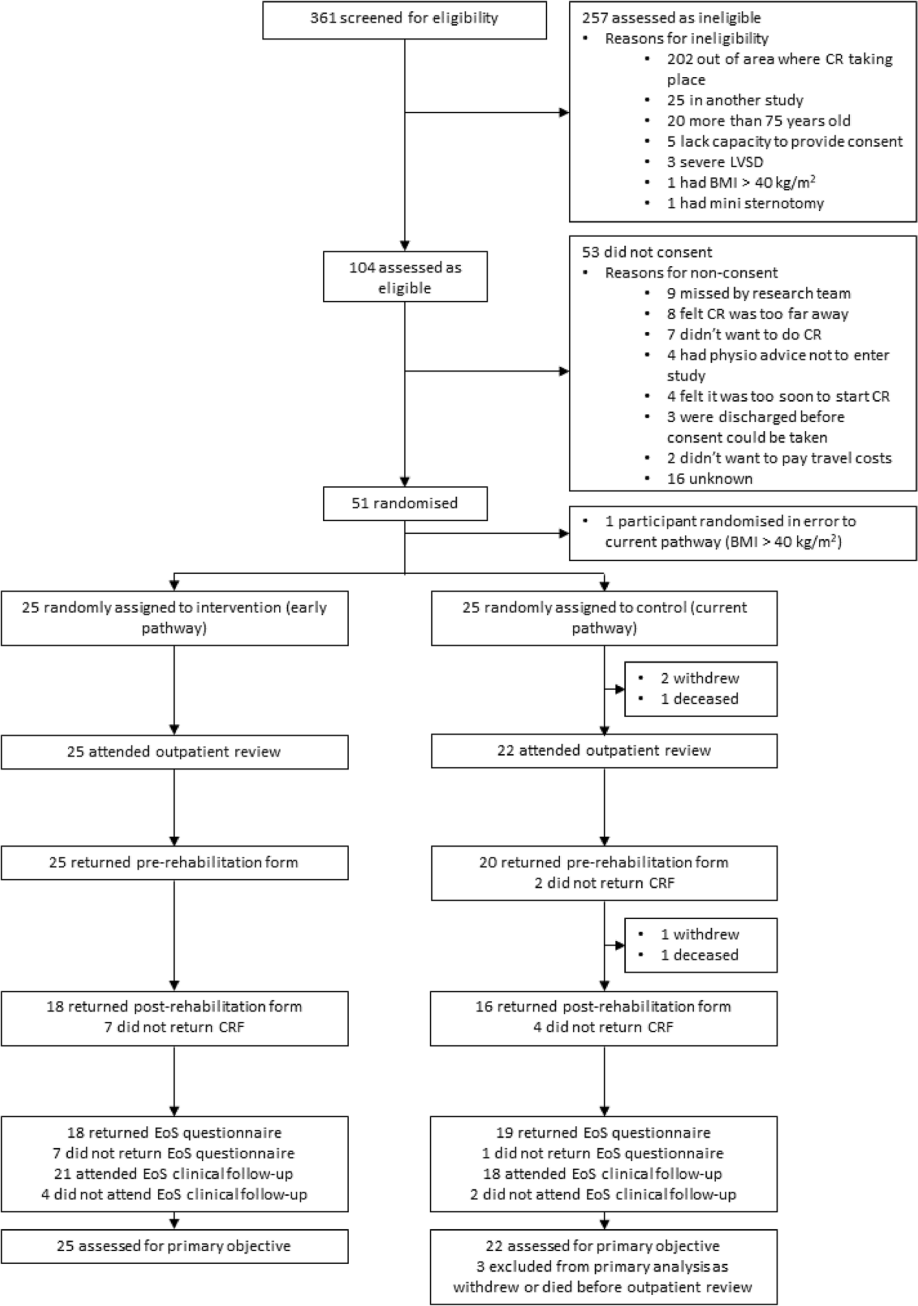
CONSORT diagram

A CONSORT diagram [19] shows the flow of participants through the trial (Fig. 1).

The trial was open to recruitment between May 2019 and December 2019. In total, 361 patients were screened for eligibility; 257 (71.2%) were ineligible, mainly because they were living far from the study CR venues (*n* = 202, 78.6% of ineligible patients). Of the 104 eligible, 51 (49.0%) consented and were randomised (Additional Table 1).

For the 53 eligible, non-consenting patients, the most common reason for non-consent was being missed by the research team (*n* = 9, 17.0%) and CR centre being too far (*n* = 8, 15.1%).

One ineligible patient randomised in error was excluded from summaries, leaving 50 participants (25 randomised to each group). Average recruitment rate was 3.8 patients per site per month and the recruitment rate 50/103 (48.5%, 95% CI: 39.11% to 58.11%).

### Participants

Table 1 shows baseline characteristics. Mean age was 63.6 years (SD 9.3) and 84% (*n* = 42) were male. The most common operation was a CABG triple bypass (*n* = 18; 36.0%) and 31 (62.0% participants had elective surgery (Additional Table 2).

**Table 1:** Baseline characteristics for randomised participants, by group and overall

	**Intervention (** ***n*** ** = 25)**	**Control (** ***n*** ** = 25)**	**Overall (** ***n*** ** = 50)**
**Age (years),** *n* (%)	25 (100)	25 (100)	50 (100)
Mean (SD)	62.7 (9.6)	64.6 (9.0)	63.6 (9.3)
Median (IQR)	61.0 (56.5,71.4)	67.1 (59.2, 71.1)	63.6 (56.5, 71.4)
Min, max	40.3, 75.3	48.1, 78.8	40.3, 78.8
**Gender, ** ***n*** ** (%)**			
Female	1 (4.0)	7 (28.0)	8 (16.0)
Male	24 (96.0)	18 (72.0)	42 (84.0)
**BMI (kg/m**^**2**^**),***n* (%)	25 (100)	25 (100)	50 (100)
Mean (SD)	29.6 (3.7)	28.2 (4.3)	28.9 (4.0)
Median (IQR)	29.5 (26.4, 32.1)	28.0 (24.6, 30.7)	28.8 (25.4, 30.9)
Min, max	23.6, 38.0	20.7, 37.0	20.7, 38.0
**Pre-operative presentation**			
** Angina CCS grade, ** ***n*** ** (%)**			
Class I	8 (32.0)	9 (36.0)	17 (34.0)
Class II	9 (36.0)	10 (40.0)	19 (38.0)
Class III	3 (12.0)	2 (8.0)	5 (10.0)
Class IV	5 (20.0)	3 (12.0)	8 (16.0)
Missing	0 (0.0)	1 (4.0)	1 (2.0)
**Dyspnoea NYHA grade, ** ***n*** ** (%)**			
Class I	9 (36.0)	8 (32.0)	17 (34.0)
Class II	10 (40.0)	14 (56.0)	24 (48.0)
Class III	5 (20.0)	2 (8.0)	7 (14.0)
Class IV	1 (4.0)	0 (0.0)	1 (2.0)
Missing	0 (0.0)	1 (4.0)	1 (2.0)
**Myocardial infarction, ** ***n*** ** (%)**			
Yes	9 (36.0)	14 (56.0)	23 (46.0)
No	16 (64.0)	11 (44.0)	27 (54.0)
**Coronary artery disease severity, ** ***n*** ** (%)**			
Single vessel disease	1 (4.0)	1 (4.0)	2 (4.0)
Double vessel disease	7 (28.0)	7 (28.0)	14 (28.0)
Triple vessel disease	15 (60.0)	15 (60.0)	30 (60.0)
None	0 (0.0)	1 (4.0)	1 (2.0)
Missing	2 (8.0)	1 (4.0)	3 (6.0)
**Left ventricular ejection fraction, ** ***n*** ** (%)**			
≥ 50%	20 (80.0)	21 (84.0)	41 (82.0)
30–49%	5 (20.0)	4 (16.0)	9 (18.0)
**Co-morbidities, ** ***n*** ** (%)**			
Hypertension	15 (60.0)	18 (72.0)	33 (66.0)
Diabetes mellitus	4 (16.0)	6 (24.0)	10 (20.0)
Peripheral vascular disease	2 (8.0)	2 (8.0)	4 (8.0)
Chronic obstructive pulmonary disease	1 (4.0)	3 (12.0)	4 (8.0)
Asthma	1 (4.0)	2 (8.0)	3 (6.0)
**Smoking status, ** ***n*** ** (%)**			
Never smoked	8 (32.0)	9 (36.0)	17 (34.0)
Ex-smoker	16 (64.0)	15 (60.0)	31 (62.0)
Current smoker	1 (4.0)	1 (4.0)	2 (4.0)

*BMI* Body mass index, *CCS* Canadian Cardiovascular Society, *NYHA* New York Heart Association, *SD* Standard deviation, *IQR* Interquartile ranges

Participants received surgery between 2 and 8 days (median 4) prior to randomisation. The most common operation was triple coronary artery bypass graft (*n* = 18, *n* = 36.0%). Most surgeries were elective (*n* = 31, 62.0%). Hospital postoperative length of stay was 3 to 10 days (median 5). In general, group characteristics were well balanced, although there was a higher proportion of males in the intervention group (96.0% vs 72.0%) and a lower proportion of participants with pre-operative myocardial infarction (36.0% vs 56.0%).

### Fitness for delivery and completion of cardiac rehabilitation

One participant was not fit for CR and one required two reviews before being declared fit (both control: Table 2). Time between surgery and the first outpatient review was approximately 3 weeks longer in the control compared to the intervention group (median 27 vs 45 days).

**Table 2:** Fitness for cardiac rehabilitation at outpatient review and time between surgery and first outpatient review

	**Intervention (** ***n*** ** = 25)**	**Control (** ***n*** ** = 22)**
**Declared fit for CR,** *** n*** ** (%)**		
Yes	25 (100)	21 (95.5)
No	0 (0.0)	1 (4.5)
**Number of outpatient appointments required before being declared fit, ** ***n*** ** (%)**		
1	25 (100)	20 (90.9)
2	0 (0.0)	1 (4.5)
Not declared fit	0 (0.0)	1 (4.5)
**Attended both the outpatient review appointment and pre-CR appointment, ** ***n*** ** (%)**		
Yes	23 (92.0)	18 (81.8)
No	0 (0.0)	4 (18.2)
Missing	2 (8.0)	0 (0.0)
**Time between the date of surgery and date of first outpatient review, days,** *n* (%)	25 (100)	22 (100)
Mean (SD)	26.2 (4.7)	45.6 (4.1)
Median (IQR)	27 (23, 29)	45 (44, 50)
Min, max	16, 39	37, 53
**Days between the date of surgery and date of outpatient review when declared fit,** *n* (% of those declared fit)	25 (100.0)	21 (100.0)
Mean (SD)	26.2 (4.7)	46.1 (4.9)
Median (IQR)	27 (23, 29)	45 (44, 50)
Min, max	16, 39	37, 58

*CR* Cardiac rehabilitation, *SD* Standard deviation, *IQR* Interquartile ranges

One further participant withdrew and another died before outpatient review. Forty-six were declared fit for CR (25 intervention; 21 control). On average, the pre-CR Case Report Form was completed 58.2 (SD 6.2) and 38.9 (SD 11.1) days after surgery in the control and intervention groups respectively. Participants in the control group completed, on average, 9.5 sessions of CR (SD 5.1) compared to 8.2 (SD 5.1) in the intervention (Table 3).

**Table 3:** Cardiac rehabilitation data presented by treatment group

	**Intervention group declared fit for CR (** ***n*** ** = 25)**	**Control group declared fit for CR (** ***n*** ** = 21)**
**Number of CR sessions attended, ** ***n*** ** (%)**	23 (92.0)	18 (85.7)
Mean (SD)	8.2 (5.1)	9.5 (5.1)
Median (IQR)	8 (5, 12)	8.5 (8, 14)
Min, max	0, 16	0, 16
**Attended at least one CR session, ** ***n*** ** (%)**		
Yes	19 (76.0)	16 (76.2)
No	4 (16.0)	2 (9.5)
Missing (CR booklet not returned)	2 (8.0)	3 (14.3)
**Number of CR sessions completed with active participation in aerobic circuit, ** ***n*** ** (%)**	23 (92.0)	18 (85.7)
Mean (SD)	7.7 (5.2)	9.3 (5.1)
Median (IQR)	7 (4, 12)	8 (7, 14)
Min, max	0, 16	0, 16
**Offered advice on diet during CR, ** ***n*** ** (% of those who attended at least one CR session)**		
Yes	10 (52.6)	12 (75.0)
No	9 (47.4)	4 (25.0)
**Offered advice on medications during CR, ** ***n*** ** (% of those who attended at least one CR session)**		
Yes	16 (84.2)	11 (68.8)
No	3 (15.8)	5 (31.2)
**Offered advice on exercise during CR, ** ***n*** ** (% of those who attended at least one CR session)**		
Yes	19 (100)	15 (93.8)
No	0 (0.0)	1 (6.2)
**Offered advice on physical activity during CR, ** ***n*** ** (% of those who attended at least one CR session)**		
Yes	18 (94.7)	13 (81.3)
No	1 (5.3)	3 (18.7)

*CR* Cardiac rehabilitation, *SD* Standard deviation, *IQR* Interquartile ranges

### Completion of cardiopulmonary exercise testing (CPET) at baseline

In total, 29 participants (58.0%; 14 intervention; 15 control) were approached for baseline CPET, which only 7 (24.1%; 3 intervention; 4 control) completed the test. Staff availability was the most common reason for the test not being conducted (Additional Table 3).

### Follow-up data collection

Overall, return rates were 68% or above; in general, the intervention group had a higher return rate. Attrition rates were similar between groups, with the overall estimates for participant and clinician end-of-study Case Report Forms being 26.0% (95% CI: 15.9% to 39.6%) and 22.0% (95% CI: 12.8% to 35.2%) respectively (Additional Table 4).

ISWT data was collected pre-CR, post-CR and end of study. The ISWT distance walked increased across time points for both arms (Table 4). At each time point, the average distance walked was further in the intervention group than control; however, 24 participants (13 intervention; 11 control) completed their final clinical follow-up by telephone due to COVID-19 and therefore could not complete the ISWT.

**Table 4:** ISWT distance presented by treatment group. Estimates of standard deviation presented alongside 80% confidence intervals

ISWT distance, meters	Early pathway (*n* = 25)	Current pathway (*n* = 25)	Overall (*n* = 50)
Pre-CR^a^			
* n* (%)	23 (92.0)	18 (72.0)	N/A
Mean	373.5	356.1	N/A
SD (80% CI)	171.5 (144.9, 214.7)	174.2 (144.3, 226.1)	N/A
Median (IQR)	350 (260, 540)	355 (190, 450)	N/A
Min, Max	40, 670	90, 680	N/A
Post-CR^a^			
* n* (%)	16 (64.0)	15 (60.0)	N/A
Mean	506.9	462.0	N/A
SD (80% CI)	211.9 (173.8, 280.8)	167.2 (136.3, 224.2)	N/A
Median (IQR)	525 (420, 630)	460 (350, 540)	N/A
Min, max	70, 900	180, 790	N/A
**End of study**^**b,c**^			
* n* (%)	7 (28.0)	7 (28.0)	14 (28.0)
Mean	565.7	474.3	520.0
SD (80% CI)	N/A	N/A	257.5 (208.6, 349.8)
Median (IQR)	580 (280, 750)	490 (230, 660)	540 (280, 730)
Min, max	100, 1020	230, 760	100, 1020

^a^ISWT not comparable between groups at this time point due to difference in timing of follow-up

^b^SD not estimated within treatment groups due to small sample size

^c^ISWT not obtained for 24 participants who completed final clinical follow-up due to COVID-19

Mortality and readmission to hospital were approximately the same in each group (Additional Table 5). Other outcomes collected at the end-of-study clinical follow-up are summarised in the additional materials (Additional Table 6).

Only two participants completed the end-of-study CPET, therefore summaries of the data are not presented.

### Withdrawals

There were 3 full withdrawals, all control group, at 3, 62 and 63 days post-randomisation. Reasons for withdrawal were: inability to travel for CR, recent serious illness diagnosis, and sternal wound problems, respectively. One intervention participant withdrew 58 days post-randomisation following clinical advice, after feeling fatigued.

There were two deaths, both in the control group, at 25 and 105 days post-randomisation.

### Adverse events

There were 21 adverse events: 12 non-serious adverse events (NSAE) (7 intervention; 5 control) and 9 serious adverse events (SAE) (4 intervention; 5 control). Ten participants had one or more NSAEs. Breathlessness was the most common feature, constituting a third of all NSAEs. All NSAEs were judged to be either not related or unlikely to be related to the study treatment. Eight participants experienced one or more SAEs. No SAEs were judged related to the study treatment.

Two SAEs were deaths, both controls. 30- and 90-day mortality data were missing for one and three participants respectively (all controls). Ten participants were readmitted to hospital (4 intervention; 6 control).

### Surgical site complications

Table 5 shows surgical site complications. Statistics are not comparable between groups due to difference in timing of outpatient review.

**Table 5:** Surgical site complications by treatment group

	**Intervention (** ***n*** ** = 25)**	**Control** **(** ***n*** ** = 21)**
**Wound pain (0 = no pain, 10 = worst pain imaginable),** *n* (%)	25 (100)	21 (100)
Mean (SD)	2.6 (1.8)	2.2 (2.0)
Median (IQR)	2 (1, 4)	2 (0, 3)
Min, max	0, 6	0, 6
**Hospital attendance after discharge, ** ***n*** ** (%)**		
Yes	1 (4.0)	4 (19.0)
No	24 (96.0)	17 (81.0)
**Sternum wound, ** ***n*** ** (%)**		
Healed	24 (96.0)	20 (95.2)
Infected	1 (4.0)	0 (0.0)
Partially healed	0 (0.0)	1 (4.8)
**Sternum stable, ** ***n*** ** (%)**		
Yes	25 (100)	21 (100)
**Donor site wound, ** ***n*** ** (%)**		
Healed	22 (88.0)	17 (81.0)
Infected	0 (0.0)	2 (9.5)
Partially healed	1 (4.0)	0 (0.0)
Missing	2 (8.0)	2 (9.5)

*SD* Standard deviation, *IQR* Interquartile ranges

### Health economic analysis

The complete response across all five dimensions required to score EQ-5D-5L was achieved in 98% of baseline questionnaires, 88% pre-CR, 64% post-CR and 74% at final follow-up. The resource-use questionnaire achieved similar response rates. At final follow-up we observed an increase in mean EQ-5D-5L health utility from baseline of 0.202 in the intervention group and 0.188 in the control (Table 6); however, much of the difference by allocation may be due to differences in baseline utility (0.031 more in the intervention).

**Table 6:** Quality of life scores by follow-up and treatment allocation

Allocation	Follow-up	*n*	Mean	SD	Min	Max
**Intervention**	Baseline	24	0.702	0.189	0.328	1.000
	Pre-rehab	24	0.835	0.125	0.539	1.000
	Post-rehab	17	0.908	0.112	0.595	1.000
	Final follow-up	18	0.904	0.168	0.280	1.000
**Control**	Baseline	25	0.671	0.174	0.279	1.000
	Pre-rehab	20	0.834	0.146	0.442	1.000
	Post-rehab	15	0.863	0.155	0.438	1.000
	Final follow-up	19	0.859	0.177	0.298	1.000

*SD* Standard deviation

Combining resource use data with unit costs provides an estimate of the total cost to the NHS and Personal Social Services during the course of follow-up, excluding direct treatment costs associated with CR which was the same in both arms. Results showed an average cost of £1798 across the follow-up period which was highly skewed, with a few patients estimated to have costs in excess of £10,000 (Additional Table 7). Average costs were different between arms (£1519 for intervention and £2043 for control); however, this was likely due to a small number of questionnaires. Data were also collected on days of lost paid and unpaid activities, and the cost of attending rehabilitation. As with resource use data, the responses were similar between trial arms but highly skewed.

### Process evaluation/qualitative interviews

Four participants from the intervention group and six controls were interviewed (80% male) prior to CR commencement. Eight also completed a second interview after CR. Due to low numbers, five additional intervention participants were interviewed after CR. The study had planned to interview 20% of participants (10 per trial arm), but due to lower recruitment, fewer were interviewed. The final number interviewed was proportional to the final sample size (20% pre-CR; 26% post-CR). Interviews took place from November 2019 to April 2020.

Three main themes were identified across both interviews:

1. *Recovery:* Participants described pain and discomfort post-surgery, wound tenderness and the impact of activity/clothing on their wounds. For many, these were unexpected, although gradually improved. At the second interview, only those experiencing significant events (e.g. readmission, complications) referred to their recovery.
2. *Timing and expectation of outpatient review:* Five control participants felt their outpatient review could have happened sooner; all intervention participants felt their review timing was about right.
3. *Attending CR:* Intervention participants described feeling ready for CR, while many controls felt they could have started sooner. During the second interview, no one objected to having started rehabilitation earlier; one control participant wanted CR earlier, wishing to move on and ‘feel well’ again. Many spoke very positively of their CR experiences. They felt in a safe environment, with input from a health professional who was providing encouragement, reassurance, and advice when physically pushing themselves, which built confidence.

Two of the 3 research nurses who supported the trial across the 2 study sites were redeployed during the COVID-19 pandemic, so it was not possible to hold the planned staff focus group meeting at the end of the study. The research nurse that was available was interviewed and was supportive of the trial but felt study processes around study information provision could be streamlined to help recruitment. Identification of participants was challenging as it was not always possible to speak to them about the study before their operation. Similarly, when a patient was fit enough to be approached post-op, this often coincided with their discharge.

## Discussion

To our knowledge, this is the first study looking at the timing of post-operative follow-up and CR after cardiac surgery with a health economic evaluation component. It is also the first study to determine the feasibility of an RCT delivering outpatient review and CR after 3 and 4 weeks respectively. The key finding is that recruitment and retention rates show that it would be feasible to undertake a full-scale trial subject to some modifications to maximise recruitment. While there appeared to be a signal indicating the intervention group benefitted from an increase in ISWT distance, this has not been shown definitively. This study illustrates the acceptability of having outpatient review and CR earlier than current standard practice. No interviewed participants in the intervention group objected to starting CR earlier.

With recruitment slower than anticipated, the sample size target of 100 participants randomised with 70 in the final analyses was not achieved. A large proportion of patients resided far from the study CR sites. The study sites are tertiary centres, and instead of using CR programmes at referring hospitals, for the purposes of this trial, to allow appropriate study oversight, we used the tertiary centre CR teams.

Over the past decade, studies have only investigated the effectiveness of early CR following sternotomy [20, 21]. Positive outcomes for cycling and walking have been reported in the first week following surgery which have not impacted on infection or healing rates [20, 21]. There is a recently published UK single-centre, non-inferiority RCT (SCAR Trial) which evaluated early CR initiation 2 weeks post-sternotomy compared to conventional 6 weeks post-sternotomy, in 158 individuals [22]. The study reported a higher dropout rate (22% in the early group vs 29% in the usual care group) than anticipated (15%). Dropout due to medical reasons was higher in the early group (41.2% vs 26.1%). Although they reported an increase in 6-min walking distance that was non-inferior to standard care, the authors were cautious in concluding that; with “appropriate precautions” CR can be started from 2 weeks after sternotomy. It is notable that patients did not have prior clinical review by the surgical team before commencing CR. Furthermore, there is no health economic evaluation of the cost-effectiveness of early CR in comparison to standard care.

### Adaptations for a full trial

To improve venue access, a full trial would include local hospital CR teams. The recruitment process will be refined to increase the time interval for identifying and consenting patients, in order to improve recruitment. In addition, the original plan was to only include coronary artery bypass graft patients; this was later broadened (with funder and ethical approval) to include other cardiac surgeries [12]. This broader eligibility would be used in a full trial from the start. Furthermore, due to logistical issues with early postoperative CPET, this would be dropped in a full trial. We will continue to collect the ISWT at pre-CR and post-CR in the full trial, as although between-group comparisons cannot be made at these timepoints, they provide valuable information on within-group trends in recovery. Finally, a future trial will have to consider any sustained COVID-19-related CR delivery changes that may have taken place such as remote patient review and home-based CR.

## Conclusion

Our findings provide evidence that a future large-scale RCT is feasible. Majority of study processes proved acceptable to participants and healthcare teams delivering the trial; we have identified procedures and assessments that may be refined for a future trial. We anticipate these will facilitate participant recruitment and minimise patient burden. The findings of a large-scale RCT would impact clinical pathways of cardiac surgery patients, and inform national and international policy about timelines of CR and return to usual activities.

## Supplementary Information

**Additional file 1: Table 1.** Monthly recruitment presented overalland by site.

**Additional file 2: Table 2.** Post-operative details of the randomised participants.

**Additional file 3: Table 3.** Reasons for not taking the baseline CPET test.

**Additional file 4: Table 4.** Estimates of the attrition rate at each time point, presented overall (when the timing of the CRF was the same for both treatment groups) and by treatment group. One-sided only CI given for 0% attrition.

**Additional file 5: Table 5. **Mortality and hospital admissions presented overall and by treatment group.

**Additional file 6: Table 6. **Other outcomes collected at End of Study follow-up presented overall and by treatment group.

**Additional file 7: Table 7.** Summary of the complete case follow-up costs by allocation and relation to cardiac condition.

## Data Availability

The data that support the findings of this study are available from the corresponding author, upon reasonable request.

## Abbreviations

BACPR: British Association for Cardiovascular Prevention and Rehabilitation
BMI: Body mass index
CCS: Canadian Cardiovascular Society
CPET: Cardiopulmonary exercise testing
CR: Cardiac rehabilitation
EQ-5D-5L: EuroQoL-5 Dimensions 5 level
ISWT: Incremental Shuttle Walk Test
IQR: Interquartile ranges
NSAE: Non-serious adverse event
NYHA: New York Heart Association
RCT: Randomised controlled trial
SAE: Serious adverse event
SD: Standard deviation
