# Supplementary material for: A randomised controlled, feasibility study to establish the acceptability of early outpatient review and early cardiac rehabilitation compared to standard practice after cardiac surgery and viability of a future large-scale trial (FARSTER)
Source: Pilot Feasibility Stud. 2023 May 11;9:79. doi: 10.1186/s40814-023-01304-3 (PMC10172724; doi:10.1186/s40814-023-01304-3)
Supplement: Supplementary file 2 — Additional file 2: Table 2. Post-operative details of the randomised participants. [file 40814_2023_1304_MOESM2_ESM.docx]

Additional table 2: Post-operative details of the randomised participants.
