# Supplementary material for: A randomised controlled, feasibility study to establish the acceptability of early outpatient review and early cardiac rehabilitation compared to standard practice after cardiac surgery and viability of a future large-scale trial (FARSTER)
Source: Pilot Feasibility Stud. 2023 May 11;9:79. doi: 10.1186/s40814-023-01304-3 (PMC10172724; doi:10.1186/s40814-023-01304-3)
Supplement: Supplementary file 4 — Additional file 4: Table 4. Estimates of the attrition rate at each time point, presented overall (when the timing of the CRF was the same for both treatment groups) and by treatment group. One-sided only CI given for 0% attrition. [file 40814_2023_1304_MOESM4_ESM.docx]

Additional table 4: Estimates of the attrition rate at each time point, presented overall (when the timing of the CRF was the same for both treatment groups) and by treatment group. One-sided only CI given for 0% attrition.
