# Supplementary material for: A randomised controlled, feasibility study to establish the acceptability of early outpatient review and early cardiac rehabilitation compared to standard practice after cardiac surgery and viability of a future large-scale trial (FARSTER)
Source: Pilot Feasibility Stud. 2023 May 11;9:79. doi: 10.1186/s40814-023-01304-3 (PMC10172724; doi:10.1186/s40814-023-01304-3)
Supplement: Supplementary file 6 — Additional file 6: Table 6. Other outcomes collected at End of Study follow-up presented overall and by treatment group. [file 40814_2023_1304_MOESM6_ESM.docx]

Additional table 6**:** Other outcomes collected at End of Study follow-up presented overall and by treatment group.
