# Supplementary material for: A randomised controlled, feasibility study to establish the acceptability of early outpatient review and early cardiac rehabilitation compared to standard practice after cardiac surgery and viability of a future large-scale trial (FARSTER)
Source: Pilot Feasibility Stud. 2023 May 11;9:79. doi: 10.1186/s40814-023-01304-3 (PMC10172724; doi:10.1186/s40814-023-01304-3)
Supplement: Supplementary file 7 — Additional file 7: Table 7. Summary of the complete case follow-up costs by allocation and relation to cardiac condition. [file 40814_2023_1304_MOESM7_ESM.docx]

Additional table 7: summary of the complete case full follow-up costs by allocation and relation to cardiac condition
